# Supplementary material for: The Transcription Factor ZmNAC89 Gene Is Involved in Salt Tolerance in Maize (Zea mays L.)
Source: Int J Mol Sci. 2023 Oct 12;24(20):15099. doi: 10.3390/ijms242015099 (PMC10606073; doi:10.3390/ijms242015099)
Supplement: Supplementary file 1 [file ijms-24-15099-s001.zip › ijms-2621917-supplementary.pdf]

International Journal of
Molecular Sciences

MDPI

Table S1. NAC transcription factor protein for sequence alignment.

Name	Species	GenBank ID	Similarity（%）
ATAF1	*Arabidopsis thaliana*	NP_171677	73
ATAF2	*Arabidopsis thaliana*	CAA52772	70
AtNAM	*Arabidopsis thaliana*	AAD17314	75
AtNAP	*Arabidopsis thaliana*	NP_564966	76
AtNAC2	*Arabidopsis thaliana*	NP_198777	62
ANAC019	*Arabidopsis thaliana*	NP_175697	73
ANAC025	*Arabidopsis thaliana*	NP_564771	74
ANAC055	*Arabidopsis thaliana*	NP_188169	70
ANAC072	*Arabidopsis thaliana*	NP_567773	70
CUC1	*Arabidopsis thaliana*	BAB20598	63
CUC2	*Arabidopsis thaliana*	BAA19529	64
SNAC1	*Oryza sativa*	ABD52007	71
SNAC2	*Oryza sativa*	CBX55846	74
OsNAC1	*Oryza sativa*	BAC53810	62
OsNAC3	*Oryza sativa*	BAA89797	69
OsNAC4	*Oryza sativa*	BAA89798	72
OsNAC5	*Oryza sativa*	BAA89799	75
OsNAC6	*Oryza sativa*	BAA89800	74
OsNAC10	*Oryza sativa*	AND61378	75
GmNAC2	*Glycine max*	AAY46122	72
HvNAC	*Hordeum vulgare subsp. vulgare*	BAJ94096	73
TaNAC3	*Triticum aestivum*	AEI00651	75
ZmNAC	*Zea mays*	AEY78612	71
ZmSNAC052	*Zea mays*	AJK30592	69
ZmNAC55	*Zea mays*	AIB04554	74
Zma000584	*Zea mays*	AKG56066	67
Zma006493	*Zea mays*	AJE59925	64
Zma054594	*Zea mays*	ALB35041	67
Zma001259	*Zea mays*	AJE59926	72
Zma003086	*Zea mays*	ALA62811	74
Zma029150	*Zea mays*	AKG56067	64

Table S2A. Results of investigation of related traits during germination under salt stress.

Lines	Germination (%)	Germination rate (%)	Root length (cm)	Bud length (cm)	Fresh weight (g)	Dry weight (g)
C01	38.27±0.60h	42.03±0.14g	1.77±0.06d	1.24±0.10b	0.38±0.01b	0.13±0.01ef
DNAC89-C-3	61.20±3.07b	64.71±2.25b	2.22±0.21abc	1.76±0.17a	0.43±0.02a	0.18±0.01a
DNAC89-C-5	55.95±0.31c	57.43±0.74c	2.32±0.09ab	1.72±0.17a	0.43±0.01a	0.17±0.01abc
DNAC89-C-7	41.71±0.35gh	46.24±0.55ef	1.90±0.01bcd	1.53±0.15ab	0.39±0.01ab	0.14±0.01def
DNAC89-C-9	50.70±0.54d	53.43±0.74d	1.90±0.04bcd	1.39±0.03ab	0.40±0.01ab	0.14±0.01def
DNAC89-C-10	43.91±0.26fg	46.03±0.87fg	2.16±0.15abcd	1.59±0.03ab	0.39±0.03ab	0.15±0.02bcde
DNAC89-C-13	47.40±1.24def	50.03±0.87de	2.00±0.02abcd	1.43±0.14ab	0.42±0.03ab	0.14±0.02cdef
DNAC89-C-15	41.79±1.10gh	44.53±1.37fg	2.21±0.02abc	1.78±0.11a	0.41±0.02ab	0.14±0.01def
DNAC89-C-18	45.25±1.61efg	46.96±1.60ef	2.28±0.25ab	1.60±0.04ab	0.42±0.01ab	0.15±0.01bcde
DNAC89-C-20	48.54±1.59de	51.25±0.89d	1.84±0.06d	1.46±0.11ab	0.37±0.02b	0.14±0.01cdef
DNAC89-C-21	42.13±2.01gh	45.33±1.64fg	2.04±0.08abcd	1.54±0.09ab	0.38±0.01ab	0.12±0.01f
DNAC89-C-25	66.67±1.11a	69.24±0.88a	2.42±0.21a	1.78±0.14a	0.43±0.01a	0.17±0.02ab
DNAC89-C-28	41.76±1.40gh	44.38±2.22fg	2.02±0.17abcd	1.60±0.27ab	0.41±0.02ab	0.16±0.01abcd
DNAC89-C-29	41.62±1.64gh	43.69±1.68fg	1.79±0.06d	1.42±0.16ab	0.38±0.02ab	0.14±0.01cdef
DNAC89-C-31	43.56±0.70fg	46.12±0.14f	1.82±0.04d	1.57±0.11ab	0.40±0.01ab	0.16±0.01abcd
DNAC89-C-33	43.09±0.93fg	45.81±0.45fg	1.85±0.17d	1.50±0.21ab	0.39±0.01ab	0.14±0.01cdef
DNAC89-C-35	44.18±1.02fg	46.84±0.06ef	1.78±0.16d	1.43±0.09ab	0.37±0.01b	0.13±0.01ef
DNAC89-C-36	41.73±1.44gh	45.14±0.99fg	1.85±0.06d	1.54±0.06ab	0.42±0.01ab	0.13±0.01ef

Table S2B. Results of investigation of related traits during germination under alkaline stress.

Lines	Germination (%)	Germination rate (%)	Root length (cm)	Bud length (cm)	Fresh weight (g)	Dry weight (g)
C01	42.98±0.72g	52.44±0.42i	1.13±0.12f	1.77±0.45bc	0.44±0.04bcde	0.19±0.03cd
DNAC89-C-3	65.81±0.35b	74.01±1.16b	2.42±0.11a	2.65±0.17a	0.55±0.04a	0.27±0.01a
DNAC89-C-5	61.07±0.82c	69.86±1.58c	2.23±0.11ab	2.66±0.24a	0.56±0.02a	0.26±0.01a
DNAC89-C-7	46.94±0.96e	55.32±0.66fghi	1.42±0.10ef	1.63±0.19bc	0.45±0.02bcd	0.19±0.01cd
DNAC89-C-9	46.84±0.72e	55.07±0.09fghi	1.52±0.11de	1.64±0.19bc	0.40±0.03cde	0.17±0.01d
DNAC89-C-10	46.28±0.42ef	54.07±1.09ghi	2.42±0.20a	1.95±0.12bc	0.44±0.04bcde	0.20±0.01bcd
DNAC89-C-13	45.74±0.62ef	59.39±0.75d	1.75±0.27cde	2.10±0.04b	0.48±0.04b	0.19±0.03cd
DNAC89-C-15	45.88±0.72ef	54.07±0.09ghi	1.41±0.10ef	1.90±0.02bc	0.41±0.01bcde	0.22±0.01abcd
DNAC89-C-18	45.28±1.32efg	52.81±0.35hi	1.70±0.02cde	1.59±0.03bc	0.39±0.01de	0.22±0.03abcd
DNAC89-C-20	43.84±0.28fg	55.12±0.14fghi	1.52±0.40de	1.56±0.11bc	0.44±0.02bcde	0.23±0.01abc
DNAC89-C-21	66.14±0.98b	69.29±0.83c	1.89±0.40bcd	1.75±0.08bc	0.46±0.01bc	0.20±0.02bcd
DNAC89-C-25	73.36±1.20a	83.13±1.01a	1.98±0.06bc	2.76±0.14a	0.58±0.03a	0.26±0.02ab
DNAC89-C-28	43.64±0.48fg	55.77±1.09efghi	1.45±0.13ef	1.62±0.20bc	0.46±0.01bcd	0.20±0.01bcd
DNAC89-C-29	44.32±1.36efg	53.77±1.09hi	1.90±0.12bcd	1.75±0.08bc	0.41±0.02bcde	0.22±0.01ab
DNAC89-C-31	46.10±0.47ef	55.98±0.52efgh	1.54±0.03de	1.51±0.22bc	0.38±0.02e	0.20±0.01cd
DNAC89-C-33	49.49±0.63d	58.31±0.85def	1.51±0.02def	1.40±0.06c	0.43±0.02bcde	0.20±0.02bcd
DNAC89-C-35	46.24±0.23ef	58.82±1.34de	1.41±0.10ef	1.66±0.19bc	0.44±0.02bcde	0.17±0.02d
DNAC89-C-36	43.71±1.15fg	57.47±2.31defg	1.36±0.13ef	1.74±0.07bc	0.46±0.01bcd	0.20±0.01cd

Table S2C. Results of investigation of related traits during germination under saline-alkali stress.

Lines	Germination (%)	Germination rate (%)	Root length (cm)	Bud length (cm)	Fresh weight (g)	Dry weight (g)
C01	33.39±0.29f	39.50±0.49g	0.81±0.10def	0.65±0.08cd	0.36±0.01cd	0.13±0.01b
DNAC89-C-3	56.42±1.17a	62.02±0.66a	1.49±0.11a	1.00±0.03ab	0.42±0.02ab	0.18±0.01a
DNAC89-C-5	45.85±0.16c	51.01±0.66b	1.21±0.03bc	1.16±0.04a	0.42±0.01ab	0.17±0.01ab
DNAC89-C-7	35.81±0.35ef	42.86±0.71def	1.01±0.03bcde	0.70±0.08bcd	0.40±0.01abc	0.15±0.01ab
DNAC89-C-9	35.84±1.06ef	40.90±0.12fg	0.96±0.05bcde	0.62±0.11d	0.37±0.01bcd	0.16±0.01ab
DNAC89-C-10	36.03±0.14ef	41.94±0.25ef	0.78±0.09ef	0.98±0.04abc	0.35±0.01cd	0.16±0.01ab
DNAC89-C-13	36.12±0.86ef	42.17±0.81ef	0.67±0.06f	0.82±0.03bcd	0.34±0.02d	0.14±0.01ab
DNAC89-C-15	35.83±0.67ef	41.66±1.50efg	1.17±0.07bc	1.02±0.01ab	0.35±0.03cd	0.17±0.01ab
DNAC89-C-18	36.85±1.17d	43.18±0.39cdef	1.00±0.08bcde	0.90±0.01abcd	0.36±0.01cd	0.14±0.02ab
DNAC89-C-20	39.56±0.60d	44.62±0.49cd	1.11±0.10bc	1.00±0.07ab	0.38±0.02abcd	0.17±0.01ab
DNAC89-C-21	37.21±1.25de	42.51±1.05def	1.19±0.13bc	0.96±0.07abcd	0.38±0.01abcd	0.17±0.01ab
DNAC89-C-25	51.43±0.98b	61.48±0.69a	1.25±0.12ab	1.00±0.17ab	0.43±0.03a	0.17±0.02ab
DNAC89-C-28	37.12±1.48de	42.86±0.30def	1.00±0.08bcde	0.72±0.26bcd	0.40±0.01abc	0.18±0.02a
DNAC89-C-29	35.53±0.64ef	41.94±1.05ef	1.24±0.07ab	0.89±0.13abcd	0.35±0.03cd	0.18±0.01a
DNAC89-C-31	37.19±1.30d	42.84±0.86def	1.13±0.09bc	0.78±0.19bcd	0.39±0.03abcd	0.18±0.03a
DNAC89-C-33	39.55±0.59d	43.71±0.54cde	0.81±0.12def	0.78±0.03bcd	0.37±0.01bcd	0.16±0.02ab
DNAC89-C-35	39.35±1.67d	45.34±0.36c	0.93±0.09cdef	0.73±0.06bcd	0.36±0.04cd	0.17±0.01ab
DNAC89-C-36	35.81±0.65ef	41.71±0.18efg	1.07±0.06bcd	0.80±0.09bcd	0.40±0.02abc	0.16±0.01ab

Table S3A. Determination of physiological parameters of T_2 transgenic lines with *ZmNAC89* gene under NaCl stress.

Lines	Relative conductivity（%）	Chlorophyll（mg·g^{-1}）	Proline（μg·g^{-1}）
C01	36.20±0.23a	29.27±0.27c	20.58±0.56g
DNAC89-C-3	31.83±0.77b	33.50±2.10ab	27.23±1.24bcde
DNAC89-C-5	30.93±0.34b	36.13±1.24a	31.86±1.41a
DNAC89-C-7	31.43±1.15b	34.47±1.21ab	22.47±0.08efg
DNAC89-C-9	32.78±0.33ab	32.63±2.12abc	25.77±0.45cdef
DNAC89-C-10	34.13±0.89ab	33.97±1.79ab	28.35±0.10abcd
DNAC89-C-13	33.04±0.98ab	32.77±1.39abc	22.52±0.02fg
DNAC89-C-15	33.05±0.69ab	32.00±0.36abc	29.97±1.84ab
DNAC89-C-18	31.65±1.01b	33.63±0.99ab	25.20±1.04def
DNAC89-C-20	32.57±0.92b	32.70±1.46abc	26.73±1.78bcdef
DNAC89-C-21	33.25±1.66ab	31.67±0.58bc	32.03±0.07a
DNAC89-C-25	30.99±0.51b	34.40±1.00ab	31.45±2.28a
DNAC89-C-28	32.21±0.62b	30.83±0.64bc	29.45±1.08abc
DNAC89-C-29	31.25±1.71b	33.93±1.51ab	29.99±0.79ab
DNAC89-C-31	32.89±1.31ab	33.73±0.84ab	23.92±1.89efg
DNAC89-C-33	32.33±0.93b	33.90±0.25ab	23.39±1.08efg
DNAC89-C-35	32.70±1.45ab	33.70±1.46ab	22.63±0.75a
DNAC89-C-36	33.31±1.25ab	33.80±0.61ab	22.27±0.89acb

Table S3B. Determination of physiological parameters of T_2 transgenic lines with *ZmNAC89* gene under Na_2CO_3 stress.

Lines	Relative conductivity（%）	Chlorophyll（mg·g^{-1}）	Proline（μg·g^{-1}）
C01	27.64±0.52a	27.20±1.60c	27.07±0.55e
DNAC89-C-3	23.55±0.12ef	32.30±0.95b	34.19±1.08a
DNAC89-C-5	22.86±0.30f	37.90±1.17a	34.22±1.08a
DNAC89-C-7	25.84±0.05abcd	29.67±1.49bc	29.25±1.39cde
DNAC89-C-9	25.87±0.15abcd	30.50±1.48bc	29.50±0.71cde
DNAC89-C-10	26.29±0.83abc	29.90±0.96bc	31.93±0.34abcd
DNAC89-C-13	25.84±0.28abcd	31.10±0.35bc	31.81±1.69abcd
DNAC89-C-15	25.99±0.93abcd	29.93±0.60bc	29.37±0.91cde
DNAC89-C-18	26.03±0.80abc	31.63±1.27bc	31.16±1.70abcde
DNAC89-C-20	26.74±0.58abc	32.77±0.76b	31.88±0.63abcd
DNAC89-C-21	24.97±0.04cde	33.63±1.60b	29.95±1.82bcde
DNAC89-C-25	23.60±0.44ef	39.63±1.53a	33.92±1.84ab
DNAC89-C-28	26.10±0.34abc	31.87±0.72b	31.80±1.69abcd
DNAC89-C-29	26.57±0.33abc	31.27±1.33bc	29.75±1.34cde
DNAC89-C-31	26.94±0.33ab	30.83±1.85bc	28.79±0.84de
DNAC89-C-33	26.11±0.13abc	31.23±2.83bc	31.95±1.80abcd
DNAC89-C-35	25.77±0.79bcd	32.10±0.87b	31.98±1.41abcd
DNAC89-C-36	26.13±0.62abc	32.63±0.90b	31.15±1.07abcde

Table S3C. Determination of physiological parameters of T_2 transgenic lines with *ZmNAC89* gene under saline-alkali mixed stress.

Lines	Relative conductivity（%）	Chlorophyll（mg·g^{-1}）	Proline（μg·g^{-1}）
C01	33.71±0.65a	19.60±0.46d	29.27±0.85e
DNAC89-C-3	29.21±0.65e	26.50±0.70a	34.78±0.87a
DNAC89-C-5	29.07±0.09e	27.43±1.22a	34.73±1.16a
DNAC89-C-7	32.54±0.44abc	21.47±1.82bcd	32.11±0.90abcd
DNAC89-C-9	31.76±0.55ab	24.13±0.73abc	30.56±1.46cde
DNAC89-C-10	31.51±0.49bcd	20.63±1.09d	30.18±0.85de
DNAC89-C-13	32.95±0.89ab	25.63±0.27ab	31.94±1.52abcde
DNAC89-C-15	32.04±0.98abcd	25.27±0.67abc	31.97±0.26abcde
DNAC89-C-18	32.01±0.75abcd	26.33±1.21a	31.06±0.91bcde
DNAC89-C-20	31.12±0.14bcd	25.73±0.12a	32.29±0.40abcd
DNAC89-C-21	31.92±0.70abcd	24.23±0.70abc	32.18±0.66abcd
DNAC89-C-25	30.97±0.08bcde	27.10±1.25a	33.21±0.65abc
DNAC89-C-28	30.09±0.73de	21.37±2.55cd	31.09±0.95bcde
DNAC89-C-29	32.33±0.68abc	24.03±2.12abc	32.61±1.15abcd
DNAC89-C-31	30.93±0.09bcde	23.73±0.92abc	31.94±0.26abcde
DNAC89-C-33	30.62±0.64cde	26.60±0.64a	32.46±0.58abcd
DNAC89-C-35	30.88±0.39bcde	23.93±1.52abc	31.41±0.34bcde
DNAC89-C-36	32.33±0.77abc	23.37±1.55abc	32.79±0.08abcd

Table S4A. SNP and haplotype of *ZmNAC89* gene.

Haplotypes	No. of lines	SNP location															
		124	142	145	181	191	211	218	244	253	375	500	624	712	719	767	885
HAP1	40	C	G	A	G	G	G	T	C	T	G	G	G	C	A	G	C
HAP2	21	G	G	G	G	G	G	T	C	C	C	G	G	C	G	G	C
HAP3	5	G	G	G	G	G	G	T	C	T	G	G	G	C	A	G	C
HAP4	1	C	A	G	A	G	G	C	T	C	C	G	G	C	A	G	C
HAP5	3	G	G	G	G	G	G	T	C	T	G	G	G	C	A	G	C
HAP6	3	C	A	G	A	A	G	T	C	C	C	G	G	C	A	C	C
HAP7	1	C	G	A	G	G	G	T	C	T	G	G	G	C	G	G	C
HAP8	1	C	A	G	G	G	T	C	T	C	C	G	A	C	A	G	C
HAP9	1	C	A	G	A	G	T	C	T	C	C	G	A	C	A	G	C
HAP10	1	G	G	G	G	G	G	T	C	C	C	G	G	C	A	C	C
HAP11	1	C	G	A	A	G	G	C	T	C	C	C	G	C	A	C	C
HAP12	1	C	A	G	A	G	G	T	C	T	G	G	G	C	A	G	C
HAP13	1	C	G	A	G	G	G	C	C	C	C	G	G	C	A	G	C
HAP14	1	C	A	G	A	A	G	T	C	C	C	C	G	C	G	G	C
HAP15	1	G	G	G	G	G	G	T	C	C	C	G	G	C	A	G	C
HAP16	1	C	G	A	G	G	G	T	C	T	C	G	G	C	A	G	C
HAP17	1	C	G	A	G	G	G	T	C	T	G	G	G	C	A	C	C
HAP18	1	C	G	A	G	G	G	T	C	T	G	G	G	C	A	G	T
HAP19	1	C	G	A	G	G	G	T	C	T	G	G	G	T	A	G	C
HAP20	54	C	G	A	G	G	G	T	C	T	G	G	G	C	A	C	C

International Journal of *Molecular Sciences*

MDPI

Table S4B. Changes of amino acids corresponding to SNP of *ZmNAC89* gene.

No.	SNP location	Amino acid changes	Base mutation
SNP124	124	R/G	GGC/GGG
SNP142	142	A/T	GAG/GAA
SNP145	145	T/A	CCA/CCG
SNP181	181	V/M	GTG/ATG
SNP191	191	G/D	TGG/TGA
SNP218	218	V/A	GTT/GCT
SNP253	253	C/R	GCT/GCC
SNP500	500	S/T	AGT/ACT
SNP712	712	H/Y	CAT/TAT
SNP767	767	G/A	TGG/TGC

Table S5. Primer pairs list.

Assays	Primer Name	Primer sequence (5'–3')
RT-qPCR	qZmNAC89-F	CTCAACGGTGGCTCCTGCTTC
	qZmNAC89-R	TCCTTGCTTTCCATGTCGC
	qActin-F	GTTGGGCGTCCTCGTCA
	qActin-R	TGGGTCATCTTCTCCCTGTT
	Bar-F	GTTGAGCAGATCTCGGTGAC
	Bar-R	GCACCATCGTCAACCACTAC
	qZm00001d014364-F	CTCTACGACGTCGGAAACC
	qZm00001d014364-R	GGTAGTTCTGGTTGATCAGTGG
	qZm00001d044546-F	TCTGAATCCCTGTCGG
	qZm00001d044546-R	CCAATGTGCCCAACC
Clone	ZmNAC89-ORF-F	CGAATTCATGTGCCCCCAACAAAGT
	ZmNAC89-ORF-R	GGAATTCTCAGCAGCAGCGCAGGCG
Plant transformation	35S-ZmNAC89-F	CACAGGGACAACACACCATAAGA
	ZmNAC89-Hsp-R	AGAAAACACTGGGGAAAGAAACA
Sequence variation analysis	ZmNAC89-1F	CCGGAATGCCCAGCCTG
	ZmNAC89-1R	GCCAGCAGAGCAGTCGTGT
	ZmNAC89-2F	TCACCGGCCGAATTCGTCGTT
	ZmNAC89-2R	CAAGCTCCAGCTACCTTCGCA

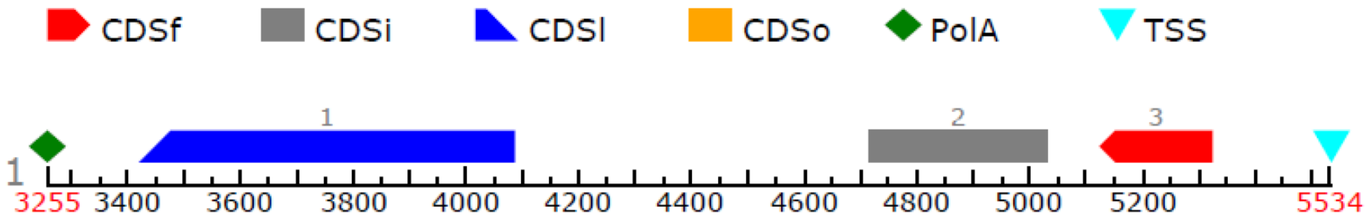

1 -		PolA	3255			0.44				
1 -	1	CDSl	3417	-	4085	53.56	3417	-	4085	669
1 -	2	CDSi	4713	-	5029	49.18	4713	-	5027	315
1 -	3	CDSf	5122	-	5323	22.01	5123	-	5323	201
1 -		TSS	5534			-4.58				

Figure S1. The gene prediction result by FGENESH. Transcription start site (TSS), first CDS (CDSf), internal CDS (CDSI), last CDS (CDSl), terminal polyA signal (PolA).

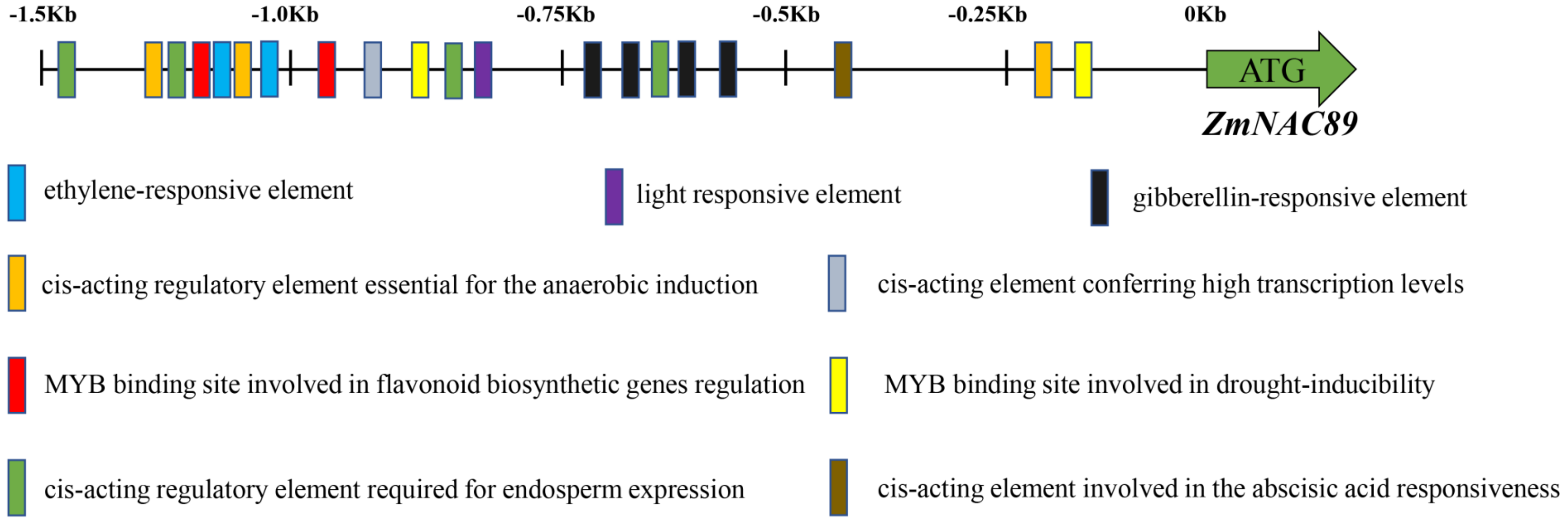

Figure S2. Analysis of flanking sequence of *ZmNAC89* gene.

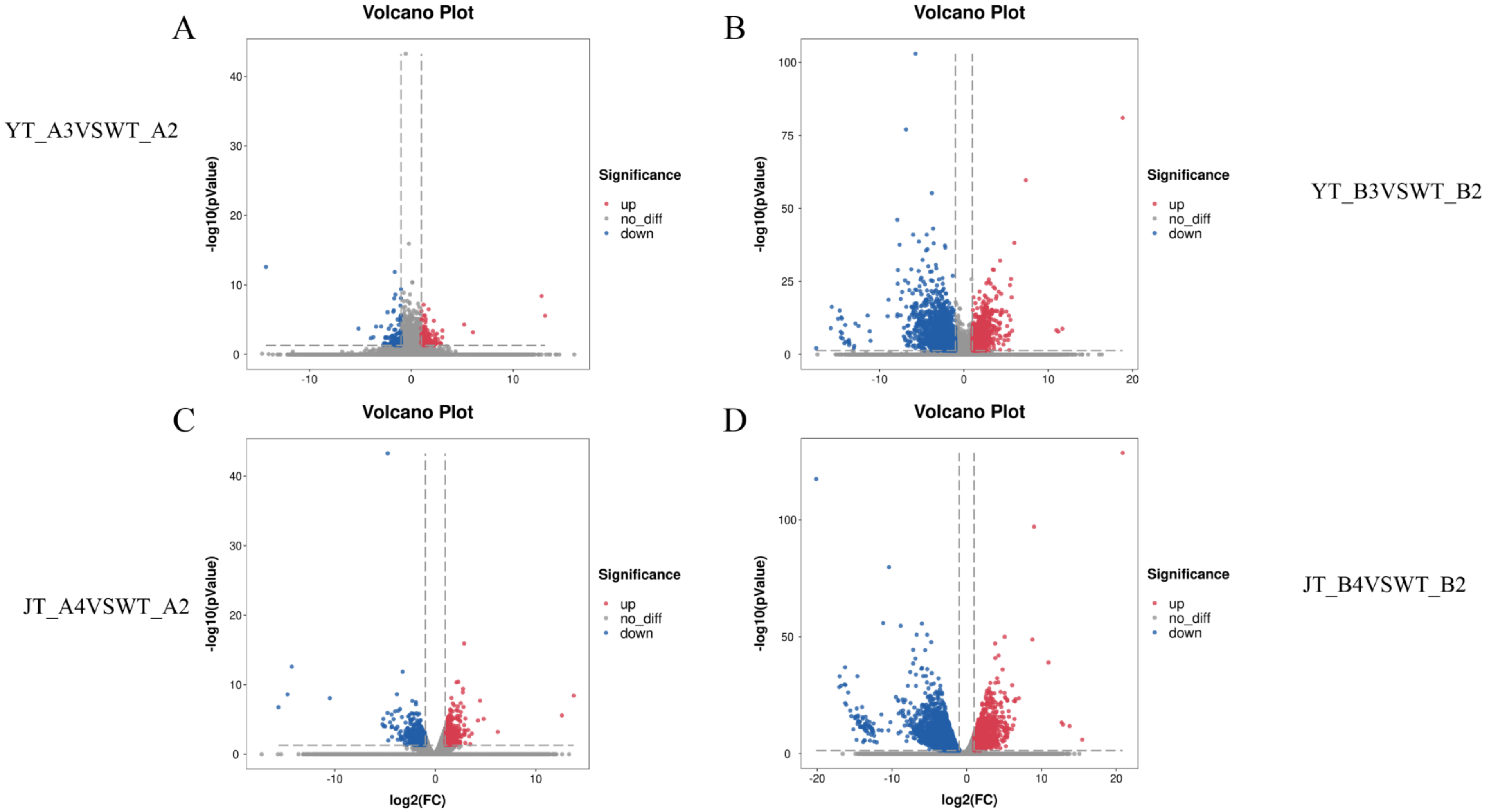

Figure S3. Different comparison group differentially expressed gene volcano map. (A) DEGs volcano map of WT plants under NaCl conditions. (B) DEGs volcano map of the transgenic *ZmNAC89* maize lines under NaCl conditions. (C) DEGs volcano map of WT plants under Na_2CO_3 conditions. (D) DEGs volcano map of the transgenic *ZmNAC89* maize lines under Na_2CO_3 conditions.

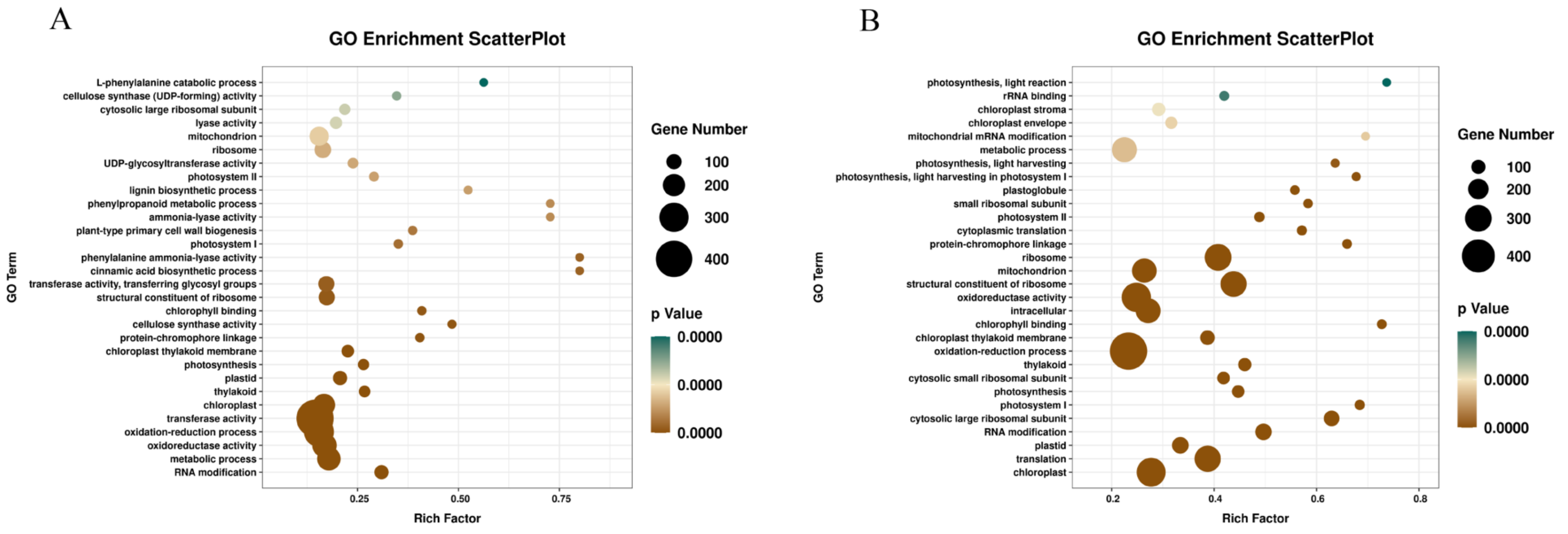

Figure S4. Scatter plot of GO enrichment under NaCl and Na_2CO_3 conditions. (A) GO enrichment analysis under NaCl condition. (B) GO enrichment analysis under Na_2CO_3 condition.
